# Children Witnessing Domestic Violence in the Voice of Health and Social Professionals Dealing with Contrasting Gender Violence

**DOI:** 10.3390/ijerph17124463

**Published:** 2020-06-21

**Authors:** Stefania Carnevale, Immacolata Di Napoli, Ciro Esposito, Caterina Arcidiacono, Fortuna Procentese

**Affiliations:** Department of Humanities, University of Naples “Federico II”, 80138 Napoli, Italy; stefania.carnevale@unina.it (S.C.); immacolata.dinapoli@unina.it (I.D.N.); ciro.esposito5@unina.it (C.E.); fortuna.procentese@unina.it (F.P.)

**Keywords:** witnessing domestic violence, gender violence, social health professionals’ competencies, intervention

## Abstract

Witnessing domestic violence (WDV) is recognized by the Istanbul Convention as psychological abuse that has dramatic consequences on the psychophysical health of children. Therefore, professionals who form the support network for WDV victims play a very fundamental role. In order to draw up useful guidelines for services dealing with WDV, and to give children more awareness of supportive settings, this study analyzes WDV in the perception of health and welfare professionals to enhance their skills and strategies for contrasting gender violence. Sixteen Neapolitan specialists dealing with WDV children were interviewed. A theoretical intentional sampling was used. Narrative focused interviews were carried out, transcribed verbatim and analyzed through the grounded theory methodology, using the ATLAS.ti 8 software (Scientific Software Development GmbH, Berlin, Germany). We assigned 319 codes and grouped these into 10 categories and 4 macro-categories. The analysis of the texts led to the definition of the core category as “The Crystal Fortress”. It summarizes the image of the WDV children as described by the professionals working in contrasting domestic violence. In this structure the parental roles of protection and care (fortress) are suspended and everything is extremely rigid, fragile and always at risk of a catastrophe. It also symbolizes the difficult role of health professionals in dealing with such children and their families. For WDV children, protective factors guarantee solid development and supportive settings help them to learn proper emotional responsiveness and expressiveness and to develop their skills in talking with adults while avoiding negative consequences.

## 1. Introduction

The Child Welfare Information Gateway [1] defines witnessing domestic violence (WDV) as psychological violence that occurs when the child witnesses domestic violence (DV) and/or intimate partner violence (IPV). Children witnessing violence against their own mother undergo psychological abuse that may have dramatic consequences for their psychophysical health [2]. However, the detection of WDV is only possible when the gender-based violence suffered by the mother has already been recognized or reported [3].

WDV assumes several forms and it can result in various severe psychological effects on the child, producing a transgenerational issue: the child may consider the abuse and the gender-based violence as something acceptable or inevitably to be repeated. Therefore, in order to avoid a likely chain of violence through generations, strong interventions in this area become necessary, both with the purpose of supporting children exposed to DV, and preventing the further development of new violence [4].

The World Health Organization [5] (p. 3) in its multidimensional vision, declares the ecological model as, “*the most widely used model for understanding violence*”, identifying violence as, “*a result of factors operating at four levels: individual, relationships, community and societal*”.

Di Napoli et al. [6] defined an ecological operational model in contrasting violence against women, following a study of four perspectives and highlights how essential it is to work synergistically on all levels involved, ensuring essential interaction among the various services in order to offer support and resources for contrasting the phenomenon of domestic violence against women.

There are various services aimed at taking charge of people involved in domestic violence: (a) national, hospital and territorial health services; (b) territorial social health services; (c) anti-violence centers and shelters; (d) law enforcement and local police forces; (e) public prosecutor at the ordinary court and at the juvenile court (civil-criminal for minors); (f) territorial institutions (regions–provinces–metropolitan cities–municipalities) (DDL 1296, 2/12/2008).

Alongside these contexts, there are associations and specialized private services that activate multi-level prevention processes and initiatives exploring the victim /perpetrator relationship, the “collateral victims” (witnesses), law enforcement and services, centers and specialists involved, who undertake networking as needed.

However, legal and social policies aimed at protecting, assisting and empowering women and bringing offenders to justice may not consider the child witnesses of violence as a direct target for services [6].

At the same time, there are many studies exploring WDV and its consequences [7,8,9,10,11], but there is little research concerning welfare and health professionals involved in the care of child witnessing domestic violence.

Furthermore, local authorities and associations have reduced budgets, leading to limitations in training, supervision and professional development for their staff members [12].

Conversely, the quality of the network fighting against DV and WDV determines the effectiveness of the intervention, both preventative and emergency [6]; therefore, reflectivity, positionality [13,14], listening and understanding all become very important. skills for welfare and health professionals operating for the prevention and contrast of gender and domestic violence.

Above all, in addition to investigating the gaps and resources of specialized services, there is a need to explore the contextually dependent meaning (representations) that “inform” these intervention and prevention settings and how they themselves are “informed” and “formed”.

This fosters much more awareness in prevention and intervention contexts in which professionals who work in health and child welfare institutions can reflect on their own thoughts and knowledge and become involved more responsibly.

Therefore, the following study based on social constructivism [15,16] and symbolic interactionism [17,18] within an ecological frame and through the exploration of shared meanings in health and child welfare contexts pursues the ultimate goal of tracing an organizational guide for intervention and prevention actions aimed at taking care of the phenomenon.

### 1.1. Literature Review

#### 1.1.1. Witnessing Domestic Violence (WDV): Consequences for Children

Research suggests that while many parents avoid involving their children in conflicts, the children commonly hear, see, and are aware of them [8,19,20].

The Istanbul Convention [21] recognizes that children are victims of DV, also as witnesses of violence in the family.

“Effective co-operation between all relevant state agencies, including the judiciary, public prosecutors, law enforcement agencies, local and regional authorities as well as non-governmental organizations and other relevant organizations and entities, in protecting and supporting victims and witnesses of all forms of violence”.

(p. 31)

WDV assumes several forms and causes a wide range of psychological and behavioral effects [9,22,23,24].

Research has detected that parents themselves underestimate the phenomenon of child witnessing violence, while children are fully aware of it [25,26,27].

Literature has widely shown the WDV psychological consequences on children, resulting in post-traumatic disorders, depression, self-depreciation, problems in school and concentration, low self-esteem, drug or alcohol abuse (in the long term) with a general compromising of individual growth, wellbeing and relational abilities [28,29,30,31].

Moreover, children witnessing domestic violence emotionally often develop guilt and disorganized behaviour that, in many cases, are also characterized by gender stereotypes [32,33]. They can, in fact, experience gender-based violence and abuse as acceptable, inexorable and/or to be inevitably repeated, activating a relevant risk on a transgenerational level.

Children who regularly witness violence can emulate the aggressive behaviour by adopting a violent one (especially for males) themselves, by identifying themselves with the perpetrators; they can also emulate remissive behaviour, assuming the danger of suffering violence (especially for females, in identification with the mother) [4].

Regular exposure to DV often affects the affective relationship between children and the non-violent parent (who is often the mother/direct victim) [34].

Therefore, interventions aiming at the recovery of the relationship between the child and the non-violent parent are necessary.

Nevertheless, not all children who witness IPV in domestic contexts have negative consequences [9,35].

In fact, individual resilience factors pertaining to the child, family or interpersonal factors, and cultural, ethnic, or community factors have been identified by Gewirtz and Edleson [36].

In particular, Miller-Graff et al. [37] showed that the way parents act in relation to the child mediates the relationship between IPV and child’s functioning and health.

Indeed, having stable adults in the child’s life helps them to learn proper emotional responsiveness and expressiveness, allowing them to have subsequently higher social competence and cognitive abilities [38].

Children may perceive their father’s violence against their mother in different ways [39]; they may believe that it is a loss of control caused by anger or they may think the victim provoked the perpetrator, or believe the perpetrator was unhappy, mean or jealous [40].

Therefore, talking to children about violence changes the way they think about conflicts between parents and DV [11].

At the same time, offering a child a support helpline network that is responsible and aware of shared meanings, in which he can express his emotions, could be a protection factor.

#### 1.1.2. Taking Care of Children Witnessing Violence: Challenges and Obstacles

The Convention of Istanbul introduced WDV as a kind of child abuse, requiring the need for appropriate interventions in this field.

Therefore, specific training in DV and its management becomes necessary in working with all the victims of violence, perpetrators and children, in order to offer a network of appropriate prevention, sensibilization and intervention [6,41].

At the same time also, the care and listening to these workers must be ensured, both to avoid developing symptoms related to working daily with experiences of trauma and stress, and to improve the quality of their intervention [6,12,42].

They are bearers of social and cultural correlates intertwined with their individual stories and relationships, including the therapeutic one; giving voice to the meanings they attribute to WDV means making space for everything they bring to their WDV vision, but it also means investigating resources and obstacles of the contexts of intervention with the aim of improving them [6,43,44,45].

While interest in the meanings shared by welfare and health professionals working with women victims of violence and perpetrators has grown recently [6,43,44,46] the representations and feelings of practitioners working with WDV children still remain in the shadows. Social and health professionals are not totally aware of what it means for a child to enter and live in the arena of domestic violence.

Research describes the perceived beneficial effects of the child talking about his or her experience of witnessed violence [11,47,48].

However, not all children talk freely about their experiences of domestic IPV, both in family and friendship contexts and in those supporting services [49,50]. This depends on the relationship with the perpetrator, the feelings of self-blame [51], and the effective opportunity or a perceived valid purpose for speaking and having an individual to tell [52,53].

Despite this, Cater [49] has shown that talking about experiences of violence in counseling and supportive contexts allows the child to feel relief and more relaxed in talking about it.

The supportive approach, based on the interpretation of maladaptive personal and relational themes in order to create a clinical setting and therapeutic alliance [54] represents, according to Horvarth and Luborsky [55], one of the predictive factors of the positive outcome of the therapeutic intervention.

However, it requires specific training and meta-reflecting competence in the care.

In this context, this study explores the representations and experiences of violence that health and welfare professionals “bring” to their social and health professional experience with children and in their listening/management of DWV.

### 1.2. The Research

The research, in a constructivist and symbolic perspective, investigates relationships and contextually dependent means (representations) that “inform” health and welfare professionals who work with people involved in DV and guide their actions. The exploration of their emotional, representational, symbolic and relational dimensions, which are shared and brought into professional contexts, are fundamental for being explicit, because they structure service relationships by “directing them” [13].

Understanding “how” and “with what” people interact allows for an awareness process that enables the individual to “think emotions” [56,57], creating conscious, responsible and sustainable processes of change and containment.

## 2. Materials and Methods

### 2.1. Aims

In addressing the settings for DV and children witnessing violence and in order to make them more aware and responsible, this research aims to: investigate the dimensions of WDV in the perception of health and social practitioners; give voice to the actors and victims of violence, as perceived by the professionals working in the prevention and intervention systems, shedding light on the childhood dimension, as further indirect victims. Moreover, it explores the resources of and obstacles faced by services, as perceived by practitioners, and collects their proposals.

The final aim is to draw up useful guidelines for training courses for health and welfare professionals and stakeholders dealing with DV and WDV.

### 2.2. Participants

The study involved 13 female (81.25%) and 3 male (18.75%) professionals engaged in the prevention and treatment of child victims of witnessed and/or direct DV, aged between 31 and 70 years (mean age = 50.95), both volunteers and professionals.

Participants were selected from a wide range of practitioners and specialists of health and social services dealing with DV and WDV after a careful analysis of the most relevant contexts for achieving the research objectives. The participants’ gender is not equated, but representative of the distribution of gender in the contexts of taking care of DV and WDV in the Neapolitan services; 62.5% of them have over 15 years of experience in the field of violence (mean = 18.31).

They have different professional roles: psychologists, psychotherapists, social workers, CTU (Office Technical Consultant), healthcare managers, emergency surgeon (see Table 1).

Participants were selected among cultural, health and social workers in the following contexts: health services (Operational Unit of Clinical Psychology, Naples Health Services, District 31, Operational Maternal and Child Unit, A.S.L. NA 1), social services (Minor and Family Service of the Municipality of Naples, Center for Families, private practices of clinical psychology).

They were selected because they work with children, focusing their attention on their emotions and their containment.

Rather than considering the victim /perpetrator dynamic, they are in favor of interventions with both victims and perpetrators, concentrating on the whole family in which the child is the victim to be supported. They work with the family and take it into therapy, or other times they provide medical or psychological treatment for the children.

Most of them activate creative workshops to give voice to the experiences and emotions of the children, allowing them to express themselves in a containing context.

Following the Grounded Theory Methodology (GTM), an initial purposive sampling was used, followed by a theoretical intentional sampling [58,59,60,61]. This is a non-probabilistic procedure that selects individuals with the purpose of ensuring adherence of interpretations to the reality of the phenomenon and of deepening the analysis of emerging data [58,62].

Indeed, during the first step participants were selected with the research purpose in mind and, after an initial analysis of interviews, in the second step we expanded the group of participants following the theoretical saturation criterion.

It is this criterion that guides the sampling process during the GTM and allows researchers to judge when to stop.

It is: “The point in analysis when all categories are well developed in terms of properties, dimensions and variations. Further data gathering and analysis add little more to the conceptualization, although variations can always be ‘discovered’” [58] (p. 263).

Participants were very varied to guarantee coverage of services effectively exploring DV.

They were contacted by phone to schedule appointments, after being selected in the contexts of family violence services of Naples area.

We introduced ourselves with a short presentation of the research and its purposes, and they readily agreed to answer the questions.

#### Ethical Issues

Ethical approval for this study was granted by the Ethical Committee of Psychological Research, Department of Humanities at University of Naples “Federico II” (CERP 15b/2019-15/3/2019).

Audio recordings were encrypted, password-protected and stored on a secure password-protected server, with the anonymized interview transcriptions.

Consent forms were stored in a locked archive, and a confidentiality protocol was followed by researchers. Participants were informed that they could consult their consent to participate in the study at any time.

### 2.3. Procedures and Methodology

A narrative focused interview [63,64,65] was used. It was based on 3 dimensions of interest, highlighted through a reference grid, in accordance with the most recent literature.

This research procedure offered the researcher the chance to develop themes of interest and also allowed the interviewee to propose topics of interest, following their flow of thoughts.

The interviews’ thematic areas were:

(a) Representation of the experiences of children witnessing violence in cases of DV;

(b) Intervention in DV and WDV: perceived resources, obstacles and suggestions; and

(c) Stories of violent scenes.

The thematic categories were identified according to some sensitizing concepts [18] and related to the aims of the study (to examine the experience and representations of health and welfare professionals dealing with cases of child witnesses of violence).

The interviews, carried out at the interviewees’ workplace or at the Department of Humanities of the University of Naples “Federico II”, were transcribed verbatim.

They were conducted in a quiet and reserved environment and each lasted from 30 min to 2 h, with an average of 50 min.

According to the constructivist approach, the analysis of the texts was activated through the grounded theory methodology [58]; a methodology that allowed us to derive more information from the data [66] and to discover theories and concepts starting from the empirical research process, trying to disregard existing theories.

The qualitative work on the texts intersected observation and theoretical elaboration in a bottom up process characterized by circularity and by the GTM steps.

The coding procedure was divided into 3 coding phases.

The activated coding types were as follows:

(a) Open coding. Sensitizing elements (parts of texts defined as *quotations*) were selected in the texts to which codes were assigned. Subsequently, the codes were grouped into categories, through a work of progressive theorizing.

(b) Axial coding. Sometimes named thematic coding represents the coding that concerns the definition of relationships among the various codes, during the open coding.

(c) Theoretical coding. Proceeding to a higher level of abstraction, more generic overdetermined categories and macro-categories were created, allowing the formulation of the new theory. They allowed research to approach the formulation of the final theory.

The codes were continuously compared in order to identify common and specific conceptual categories to be organized in overdetermined groups through reduction/condensation and revision. In this process, the researchers’ consent criterion was followed.

In the final phase a “Core Category” was created; a new central category detached from the data, around which connections intersect. It “tells” of the texts and interpretations made during the whole process. This was an inferential insight that allowed us to find a new meaning in the data that was not explainable through categorizations that had already been carried out.

## 3. Results

In the textual material 319 codes were assigned in the open coding phase and subsequently grouped into 11 categories and 4 macro-categories. The macro-categories are shown below and they concern the issues that emerge from the sensitizing concepts encoded in the interviews. The interviewees’ words are shown in quotation marks, testifying to the topics highlighted.

Results give voice to the helpline staff that provide support to victims of DV, perpetrators and families and show their representation of the WDV phenomenon and their work experience and proposals.

### 3.1. WDV in the Experience of Health and Welfare Professionals

This macro-category includes the representations of the interviewees about WDV and the effects of the violent relationship on children and parenting in cases of IPV in family contexts.

The categories here are:

(a) A multi-layered collusion.

At the family and socio-cultural levels this expressed the “normality of violence” in patriarchal culture in which,

“If you ask a child who grew up in a violent family why he slapped his classmate or why his father spat in his face in a moment of anger, he answers ‘I don’t know’, because it’s like asking the reason for something that is done ‘automatically’, because it is always done in this way, there are no explicit explanations”.

(Psychotherapist and Technical Office Consultant, Court of Naples and private specialist, M, 70 years old)

Interviewees described a cultural substratum that shapes and informs, and is formed and informed by a patriarchal culture that is reiterated within the family relational dynamics. It conveys the logic of gender inequalities (imbued with violence in its essence) and becomes rigid and destructive in cases of violence.

Moreover, professionals who work in contexts that children live in daily, and who are delegated to recognize the first signs of violence (schools, babysitters, sports teachers) are not trained to recognize the children’s experience of domestic violence [41,67].

(b) The invisibility of children who witness domestic violence.

Users of services often claim not to “consider” the presence of children in quarrels, *“We often ask ‘But were the children present?’ and only in that moment they say yes. They don’t say it in the story”* (Psychologist, Private, F, 35 years old). WDV is recognized as a multidimensional phenomenon characterized by the fact that children become “invisible” for parents in the moments of strong conflict, as if a splitting process occurs and separates the “conflict bubble” from the rest of the context.

(c) “Adultized” and exploited children.

The invisibility of children accompanies the triangulation of relationships with them. They are too often “exploited” and made part of the regular conflicts and tensions that characterize the relationships between parents.

These children become tools for the parents to injure each other, to threaten each other, and again to communicate.

Interviewees reported that these dynamics make them “adultized children”; they often have to be careful how and what they say, how to behave, trying to reconcile the image of the “parental couple”.

“They become the parents. They become the ones who protect the mother, the ones who distract the father, because they have learned to read the moment when the tension breaks out”.

(Psychotherapist and Technical Office Consultant, Court of Naples and private specialist, M, 70 years old)

According to the interviewees, these children have had to learn to recognize the moments of rising tension, trying to avoid them and being careful not to make mistakes; they have to pay more attention to the context and develop skills that make them grow up too quickly and/or allow them to develop post-traumatic symptoms.

As the same interviewee reported:

“… children don’t love dad or mom. Children love the couple. When the couple breaks up something in the children broke”.

(Psychotherapist and Technical Office Consultant, Court of Naples and private specialist, M, 70 years old)

(d) Forced deployment and post-traumatic stress syndrome: the effects of the violent relationship on children.

Children, according to the interviewees, at some point, *“are almost forced to take a position with one or the other parent”* (Psychotherapist and Technical Office Consultant, Court of Naples and private specialist, M, 70 years old).

Interviewees said that the children show a strong dread of abandonment and death for the risk that the father may kill the mother or a catastrophe may occur. They feel a strong need to take sides with one or the other parent.

However, the deployment with one of the parents does not restore serenity to the child, but rather, leads him to the forced loss of an important part of his growth; this leads him, together with everything else, to manifest a symptomatology that recalls post-traumatic stress syndrome, which is identified as the mirror of the mother’s own symptoms.

*“They* (children) *often reported nightmares in which they dreamed of the father who removed them from their mother and wanted to kidnap them. Much younger children reported having dreamed of monsters, or they recurred in graphic production and drawings. Moreover, children talked about the classic stress symptoms, such as bedwetting, lack of appetite, difficulty eating, or difficulty concentrating in school, recreational activities, the continuous request for protection from the other parent”.*

(Social Worker, Minor and Family Service, F, 44 years old)

The children’s experience of abandonment and death anxiety is the one most commonly highlighted by the participants, with their fear that a catastrophe, death, removal and loss of parents may occur.

“And they worry about the mother, because they are afraid of the blood, of the wounds and of the threats, of the shouting etc., and the fear above all. They are children who are very scared [...] so when I comfort them, I hug them, they are also a little wary of this, however, they are children who suffer a lot”.

(Social Worker, Minor and Family Service, F, 44 years old)

They are children, according to the interviews, who on a behavioral level can run away, scream or take refuge in their media world (when they are older), but they present the symptoms of continuous exposure to trauma.

Emotionally, they often experience a sense of destructive guilt that cages them in “crystallized” dynamics where any small change can bring down the family structure.

“Children are present and they are also very guilty of this thing, because maybe they feel guilty about this phenomenon, because they cannot understand the dynamics. They often become the cause of the quarrel, because going out with a child, or having accompanied him to school, can unleash jealousy etc.”.

(Emergency Surgeon, ASL, F, 55 years old)

(e) Personal experiences, stereotypes and prejudices of professionals working in services dealing with tacking care of DV families.

Prejudices and stereotypes about WDV do not emerge from the texts and no interviewee claims to have personally experienced WDV in childhood.

However, they agree that we are all victims of gender stereotypes, including men.

“Even young boys are always told ’boys don’t cry or don’t act like a girl!”.

(Psychotherapist, ASL, F, 65 years old)

According to them, our culture, bringing with it the roots of patriarchy, perpetrates stereotypes and gender discrimination.

Indeed, all female participants claim to have experienced episodes of discrimination in the family and especially at work; they have suffered gender stereotypes, often recognizing them, but still suffering.

“My father always asked me to prepare the table for meals and when I rebelled and I asked why me and never my brother, he said it was not a man thing!”.

(Psychotherapist, ASL, F, 65 years old)

### 3.2. Psychological and Behavioural Effects of Witnessing Violence on Children

This macro-category includes the interviewees’ testimonies about the effects on children of daily witnessing of domestic violence.

The categories here are:

(a) Post-traumatic stress disorder symptomatology

All the participants reported symptoms of Post-Traumatic Stress Disorder, both for children and mothers.

The following symptoms were reported: hyperactivity, aggressive refusal towards the outside world, violence towards siblings or classmates, sleep disturbances, request for protection by the abused parent, enuresis, loss of appetite, stress, concentration difficulties, gastrointestinal disorders, learning and attention disorder, stuttering.

As a social worker explained:

“They are children who certainly show great suffering, or closing up, and therefore, in practice they are extremely rejecting towards the outside world; they begin to give signals at school and very often it is the school that practically realizes, and brings attention to this behavior. Children carry out violent actions against their siblings or their reference companions. With parents they tend to be children who appear very split in behavior; they collude with one or the other parent and often they tend to please the stronger parent, because they are obviously afraid, especially in cases of witnessing violence”.

(Social Worker, Minor and Family Service, F, 44 years old)

(b) The psychic turmoil of children in WDV cases

The interviewees described children who have a deeply psychic turmoil and who cannot make sense of everything they see. Everything is always transient and uncertain, because situations can change suddenly; one moment everything is peaceful, while a second later a war can break out. As told by an interviewee:

“What terrifies them most? Change. Seeing, perceiving that an atmosphere has changed, that there are no longer the well-known parameters that are part of everyday life; father, mother, children who stay at home, eat, watch TV… Change that means change of tone, behavior, language”.

(Psychotherapist, ASL, F, 65 years old)

### 3.3. Difficult Parenting in Intimate Partner Violence (IPV) Cases

This macro-category describes how parenting in the cases of DV and WDV is deeply undermined. It becomes a very difficult function to carry out in violent dynamics in which the focus shifts from the children and family balance to the continuous search for control and power.

In these cases, children are described in a double meaning: either “invisible” or “motivation for change”, breaking violent dynamics, where there is awareness.

The categories here are:

(a) Parenting as a motivation for change.

Participants said that when the woman realizes the danger to which her children are exposed, the feeling of protection can take over and lead her to her “breaking point”. In these conditions she manages to leave the man.

However, according to the participants, the man, although very rarely, seeks help to stop repeating the violence when he becomes aware of the damage suffered by his children. But the professionals express great reticence regarding this last hypothesis.

Therefore, according to participants who express confidence in this factor, fatherhood can become a valuable starting point for a process of awareness.

According to the interviewees this awareness also includes suggestions for the service users: *“It is important to allow fathers to take on the child’s perspective, but precisely in the interaction with the child”* (Psychotherapist, Private, F, 70 years old).

In fact, according to the interviewees, fatherhood, understood as feeling, role and identity, can be a motivation for man to ask for help.

*“Fatherhood is a breach in which a little bit of change can be inserted. At least you can try it”*.

(Psychotherapist and Technical Office Consultant, Court of Naples and private specialist, M, 70 years old)

However, in order to activate these changes, the awareness of men and the activation of empathetic feelings towards the victims are fundamental.

*“The first effort professionals have to make is to bring people to the awareness that the children are experiencing something abominable, terrible and painful”*.

(Psychotherapist, OLV, M, 31 years old)

(b) Parenting undermined by DV

Generally, in the reported experience, mothers show strong depressive traits and a symptomatology that attests to their continuous exposure to situations of strong stress. For them, motherhood becomes a very difficult function to carry out as all their attention is focused on conflict and management of constant tensions.

*“The woman is so destroyed that she cannot manage to contain even the smallest impetuosity of the child”*.

(Social Worker, Consultancy, F, 58 years old)

For the father, on the other hand, in some cases the child could be a “trigger” for awareness processes, but for most of the interviewees, the child can be used as a tool to control women and to triangulate relationships.

Indeed, children often are involved in verbal conflicts or become objects of recrimination and/or threats that keep mothers in a state of tension.

*“We often face stories of threats and blackmail, where men often threaten to harm their children if women do not behave as they want”*.

(Psychotherapist, Private, M, 31 years old)

### 3.4. Intervention in Cases of IPV in Family Contexts and WDV: Representation of Resources, Deficits and Suggestions

This macro-category concerns how the participants consider their work context, the network of services with which they collaborate and the resources and the deficits that characterize them.

The categories here are:

(a) Disruption and lack of communication among specialized services.

As also analyzed by Autiero et al. [45], in the perspective of the WDV, the interviewees highlighted a lackluster dialogue among the services for women, those directed to men and those that support children.

*“There is still no integration between those who work with women, perpetrators and those who work with children”*.

(Social Worker, Consultancy, F, 58 years old)

A further obstacle that arises in this network is the bureaucratic management of the minor; when any professional working in child welfare services want to take care of them before sending them to specialized centers, you need the authorization of the fathers, who often use their children as a tool for blackmailing the mother.

In fact, a participant affirms:

*“We have a big problem; when you have to help a child, you need the authorization of the abuser who makes it a blackmail element. And therefore, that child is suspended there, with all his traumas, with all that he has experienced ”*.

(Social Worker, Consultancy, F, 58 years old)

In the words of participants, this context has a lot of obstacles and only strong determination, good training and preparation and a solid network will allow a professional to do his job effectively.

(b) The importance of prevention and sensitization for awareness.

A particular focus is placed on the importance of activating interventions aimed at developing the metacognitive, reflective and empathic abilities of men, in order to encourage their thinking about their own actions.

*“I believe that a space for them [men] is important, because at the basis of the violence there are emotions that are not really recognized, and then lead to very heavy experiences”*.

(Psychotherapist, OLV, F, 32 years old)

*“I would work on opening a channel of understanding … that would allow the man to understand the pain it causes on his partner, but also on his children, right?”*.

(Social Worker, Consultancy, F, 58 years old)

Prevention is considered the best form of intervention. Given the strong cultural roots of the phenomenon, a slow but effective and continuous cultural change is desired.

Therefore, multiple approaches are needed. According to participants in childcare, communication is a very important tool: a dialogue made up of forms of expression that respect the history of the child. The interviewees highlighted the importance of offering children and young people various expressive channels such as drawing, story-telling, role-playing games, etc. in order to share their emotions and the meaning attributed to their family experiences with them.

*“I lead music therapy groups with children here at the family center and we often make them draw, even while colleagues are sitting with their parents. Suffering experiences really emerge and children can say many things”*.

(Psychologist, Center for Families, F, 57 years old)

Moreover, at the same time, in an ecological context, much importance is placed on the training and care of services professionals.

*“I think that having care and respect for the workers of these Service is fundamental. It would take more attention, more funds and also more moments of comparison and training, also with other realities and other countries”*.

(Psychotherapist, OLV, F, 66 years old)

### 3.5. The Core Category

**Figure 1:**
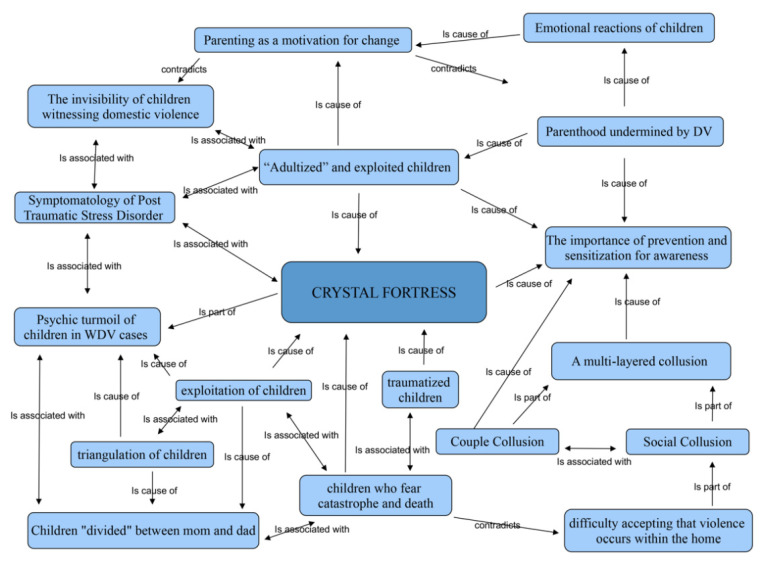
Network of “The Crystal Fortress”. This figure describes the relationships among the Core Category and significant elements that emerged from the analysis of the texts.

The analysis of the texts and categories led to the definition of a *Core Category* and to the visualization (see Figure 1) of all the conceptual networks and contents expressed by the interviewees.

The Core Category, which explains meanings, has been called *“The Crystal Fortress”*; a concept that gives shape to the individual, relational, collective and organizational dynamics that characterize the WDV and IPV among professionals who work with children witnessing violence.

The stories told by our interviewees reported a childish imagination that paints the family relational context as a crystal fortress; it is an image that expresses a sort of oxymoron in which the strength and endurance of the rough fortress are in opposition with the weakness and fragility of the elegant crystal.

The fortress represents a place built to defend oneself, a strong but vulnerable place as it is made of crystal. It is not a home, but a refuge. In the child, as the textual material revealed, it represented their “shield of strength” which they would have to create through thoughtful gestures, control of words and actions and the renunciation of their childhood innocence.

“Don’t move” seems to be their motto, in a context of defense and protection, which however is made of crystal and is continuously exposed to the risk of shattering at any time. The risk of immediate conflicts never leaves the child and is lurking behind every word, gesture or expression.

In this image the strength of the fortress is cancelled out by the material it is made of; a material as beautiful as it is fragile, as transparent as it is overshadowing.

It leaves very little space for free and carefree dreams and leaves much space for children’s greatest terror: witnessing a catastrophe that takes them away from their parents or, even worse, witnessing their destruction.

Therefore, the core category describes a family environment that may look very strong and powerful, but actually, is only fragile and needs to be carefully addressed and maintained; a family where the child has to take care of the relational environment, parents and relatives.

This emotional climate is not peaceful and children feel that an emotional storm could arrive at any moment. Therefore, the crystal fortress is meant to express on one hand the apparent strength of this family (the fortress), but on the other it emphasizes the risk of its collapse. 

The same image of the rigid and fragile fortress also describes how the professionals themselves experience their work context; a place where they find many obstacles and have few resources. They have to put a lot of effort into their work and often seek ploys in order to ensure people’s care, safety, support and sustainability of all this.

The isolation in which services often work and the limited dialogue between them creates the weakness of the system.

## 4. Discussion

The analysis of the texts shows family conflicts reported by the health professionals as “bubbles” which are split from reality, in which parents lose sight of their children while they are present at the clashes.

There is a dread of change and separation that are experienced with a deadly note, in an atmosphere of fragile balance and rigid tension.

WDV, defined in its complexity, recalls very difficult parental roles; fatherhood, understood both as an emotional bond and as confirmation of a gender identity that strengthens masculinity in its patriarchal meaning, is reported both as a risk factor and as a motivation for change.

In addition, as reported, sometimes men, especially in the first months of pregnancy, feel threatened by a “third” (the child), who looms in the relationship activating violent dynamics, as described by many women to the services [44].

In the second case, fatherhood, even though seen with a lot of skepticism, can become a factor of change for the violence perpetrator, as it could lead to change when he is put in the child’s shoes and understands the child’s suffering and pain.

Motherhood, on the other hand, instead is imbued with traumatic experiences due to the continuous exposure to stress, threats and attacks that lead the woman to manifest depressive episodes and post-traumatic stress disorder symptoms, confirming literature [68,69,70].

All this gives strength to the threats of the man who, by denigrating the woman, often accuses her of not being a good mother and threatens to take the children away.

These dynamics often also involve the contexts of the intervention and make their work really difficult. They become serious obstacles in the therapy process and children are always suspended in an interminable time of tension and fear.

Despite this aspect, the motivations for change, both for men and for women, can be found in parenting, but also in a strong awareness of the violent relationship, and this often takes place only through a path of support.

In these circumstances the role as a professional is also very difficult because of this context is extremely sectorial and disintegrated. Giving support without receiving it by any organizational system becomes a very weighty challenge to be faced by practitioners.

Indeed, the crystal fortress also represented the organizational structure in which the participants worked: a world of resources, protection and support in which accessing these functions is not easy.

According to participants, services dealing with the child are a world in which the urgency of supportive intervention and the emotional containment of the child runs up against rigid bureaucracy (there is the need of the father’s consent for intervention directed to the child) and a family history that overwhelms the child in a vortex of conflict and violence.

Furthermore, in the services network the rigidity (metaphorically that of the crystal) is an obstacle to the dialogue between the services and it is also obscuring the potential of different services (metaphorically the beauty of the crystal).

Rigidity is also in the experience of professional. WDV child has only recently entered in their professional domain and, they too, are unable to host these children; talking about their personal life they do not express memories or stories of their childhood related to domestic violence; their reflexivity beyond the fortress is to be increased or at least to be further investigated.

### Limitations and Future Perspectives

The generalizability of these results is limited because it is a qualitative and situated study, aimed at identifying specific attitudes and views of professionals dealing with WDV.

Indeed, the group of participants was not gender balanced and it was non-probabilistic, but representative of the distribution of gender in the contexts of the WDV care in the Neapolitan services.

The results are also correlated to specific geographic locations and to a specific purposive sample. This makes it necessary to verify whether our results are useful for other research contexts.

In order to examine the data emerging from the analysis more in detail further applications are then required.

## 5. Conclusions

Violent relationships in family contexts are depicted as rigidly based on power and control that hide a fragility and inability to manage stress and conflicts.

In these dynamics, children, as represented by professionals renounce the parental couple by positioning themselves with the mother or the father, and they bear the signs of this split in their symptomatology. It mirrors that same splitting that prevents parents from “seeing” them during episodes of violence.

Their invisibility is often “brought” by parents into services, similarly to the invisibility of IPV in families, because of a culture that is still reluctant to recognize the violence of gender stereotypes within domestic walls [71,72,73]. They deprive people of an opportunity for redemption in their stories.

Confirming Autiero et al.’s study [45] the same splitting mechanism characterizes the relationship between the various specialized and general services directed to families and their members where again the child is not a direct target.

Therefore, the dialogue between the various services becomes even more important and allows the activation of integrated empowerment paths that help all the family members.

According to the study, in working with child witnesses of violence, it becomes important:

1. To help parents become aware of the destructive dynamics and splitting mechanisms that activate and overshadow the child.
2. To work on parental functions, activating a dialogue between parents and children that reassures children and help them in reconstructing the sense of their experience and bring down the crystal fortress that is harmful to their growth.
3. To use a supportive and containing approach that allows the child to express his/her thoughts, fears, emotions and turmoil.

In this vein, children should be encouraged to talk about their emotions and experiences, and a protected space to do this has to be provided. Each child should have the opportunity to express themselves in the times and ways most appropriate to themselves and their history.

Participants said they had worked with the children by making them draw or express their feelings. Meanwhile, they also claimed that creative interactions offer them the chance to express themselves and to create a containing context in which children entrust adults with the heavy worlds of experiences that they cannot manage.

Dialogue has to be activated between all the actors; in the couple, between parents and children and among the same services directed to DV, on several levels, especially on the meta-reflective one.

An aware and responsible dialogue to build virtuous networks, to bring down fortresses and free DV families from their isolation.

Therefore, the care and listening of the helpline staff is fundamental, in order to be better-prepared and more capable to create healthy and emotionally sustainable spaces and support.

According to Di Napoli et al. [6], an integrated intervention is needed, also in schools with children and young people involved in families characterized by DV; this can activate a network capable of co-constructing a “fortress” made of solid rocks and a solid network of positive relationships to which to entrust experiences that would make people weak and to create a conscious and sustainable force over time.

Furthermore, it will be useful to involve professionals who work in contexts of children’s daily lives (school teachers, babysitters, sports trainers) and who are not trained to recognize the children’s experience of domestic violence [41,67]. Their mission should also be to recognize the first signs of violence on children.

## Figures and Tables

**Table 1:** Participants’ data. This table illustrates some information about participants.

Features	Percentages/Frequencies
Gender N %	13 F 81
	3 M 19
Professional Role %	69 Psychologists and Psychotherapists 25 Social Workers 6 Doctors
Work Context %	31.25 Center for Families 18.75 Health Consultants 18.75 Private Professionals 12.5 OLV (Project “Oltre La Violenza”) 18.75 Other
Years of Service % (range)	18.75 (1 ≥ 5) 0 (6 ≥ 10) 0 (11 ≥ 15) 81.25 (>15) Mean 27.5
Years in Dealing with Violence % (range)	6.25 (<1) 12.5 (1 ≥ 5) 18.75 (6 ≥ 10) 0 (11 ≥ 15) 62.5 (>15) Mean 18.31
